# Pervasive Refusal Syndrome in Autistic Spectrum Disorder

**DOI:** 10.1155/2018/5049818

**Published:** 2018-06-07

**Authors:** Emily Claire Bond, Rosalind Yvonne Kirton Oliphant

**Affiliations:** Ferndene, Child and Young People's Inpatient Unit, Moor Road, Prudhoe, Northumberland NE42 5PB, UK

## Abstract

Pervasive Refusal Syndrome (PRS) is a rare child psychiatric condition. We describe a case of PRS in a 9-year-old boy with a diagnosis of Autism Spectrum Disorder (ASD) presenting with severe weight loss due to extreme restriction of food and fluids. Other prominent symptoms included total mutism, school refusal, and self-neglect. He was admitted to a specialist Child and Adolescent Mental Health Unit. We discuss the symptoms present in this case and the differential diagnosis of ASD in PRS. Although this differential has briefly been considered one in previous case, there have been no reported cases of PRS with a prior diagnosis of ASD. We explore comorbidity and interaction of the two diagnoses. We discuss the possible impact of ASD as a predisposing factor upon the progression and prognosis of PRS.

## 1. Background

Pervasive Refusal Syndrome (PRS) is a rare and little understood child psychiatric condition. This was first described by Lask et al. in 1991[1]. Since then, diagnostic criteria were established by Thompson and Nunn in 1997 [2]. However, it does not feature in either ICD-10 or the more recent DSM-5. The diagnostic criteria are

- clear food refusal and weight loss
- social withdrawal and school refusal
- partial or complete refusal in 2 or more of the following domains: mobilisation, speech, and attention to personal care
- active and angry resistance to acts of help and encouragement
- no organic condition to account for the severity of the degree of symptoms
- no other psychiatric disorder that could better account for the symptoms.

The literature currently available regarding PRS, which is generally in the form of case reports, suggests that the typical demographics are females displaying high achieving and perfectionist personality traits, with a mean age of 10.5 years. This information also suggests that the prognosis is generally good with a high rate of complete recovery (67%). However, the process is described as ‘painfully slow' with an average of 12.8 months' length of treatment [3].

The case we discuss is an example of PRS presenting in a 9-year-old boy with an established diagnosis of Autistic Spectrum Disorder (ASD). There has been a number of different differential diagnoses for PRS discussed and a number of comorbid conditions described, including anxiety and depression [4]. Yet, there have been no other cases of PRS with ASD published, nor has there been any other evidence to suggest that this association is commonly observed in clinical practice. In one other published case report of a 4-year-old boy, also with some atypical features of PRS, the differential diagnosis of ASD or of a generic pervasive developmental disorder is discussed. However, in this case no prior or concurrent diagnosis of ASD was made [5].

Given the diagnostic criteria of PRS and the symptoms of ASD, however, it does not seem surprising to observe comorbid presentation of these conditions. Children with an ASD diagnosis can develop different eating patterns to a non-ASD population. In particular, texture and types of foods can be selective [6, 7]. There has been work into developing behavioural strategies and other treatments to address these feeding issues in ASD [8]. It is established that children with ASD can have significant language disorders. However, persistent lack of speech or mutism is generally associated with the severity of their autism and level of intellectual disability [9]. Children with ASD are known to struggle with various components of self-care such as washing and dressing [10]. Evidence suggests, especially in younger children, that these are likely to be related to sensory processing differences in the ASD population [11]. It is also known that children with Pervasive Developmental Disorders, including ASD, are more likely to present with school refusal. Again, this is more likely when the child has an intellectual disability or obsessional traits [12]. Urinary and faecal incontinence, similarly, are more common in the ASD population [13].

## 2. Clinical Case Information

Our case is of a 9-year-old Caucasian male who lives with his mother, stepfather, older brother (15 years), and sister (12 years). He has preexisting diagnoses of Autistic Spectrum Disorder (ASD) and Attention Deficit Disorder (ADD), inattentive subtype, but was not taking any regular medication at the time of presentation. He presented to our inpatient service as an emergency from a local acute hospital due to concerns regarding minimal dietary intake. He was detained under Section 2 of the Mental Health Act for assessment and treatment, due to his resistance of treatment in the community and lacking Gillick competence. He was admitted to a specialist psychiatric ward for children under 12 years. At the time of presentation his most prominent and concerning symptoms were refusing food and fluids, mutism, school refusal, and self-neglect, including refusal to engage in his personal care regime. It was the severity of these symptoms that was particularly concerning to his family and to professionals. His restriction of dietary intake resulted in severe weight loss and admission to hospital for nasogastric (NG) tube feeding, his body mass index (BMI) being less than 12kg/m2 at the time of admission (less than 0.5th percentile). His sole method of communication was typing on an iPad to his mother and his personal care was restricted to wearing his pyjamas and a coat for several days without washing or changing. His mother reports that prior to admission there were also incidents of urinary and faecal incontinence. He would not sleep in his bed but was instead sleeping on the floor outside his parents' bedroom in the “foetal position”. When family members attempted to touch him, he became physically aggressive and hit out at them. He was diagnosed with Pervasive Refusal Syndrome at this time.

In May 2015, his stepfather gave him a haircut, which he particularly disliked. This appears to have been a trigger for the subsequent observed behaviours. Following this, he became more controlling of his dietary intake and became selectively mute. In particular, he stopped talking to his stepfather and eating any food that he had prepared. This progressed in severity to the extent that, in January 2016, he completely stopped eating and drinking and became completely mute. He was admitted to the local acute hospital for NG tube insertion and intravenous (IV) fluids at this point. Blood biochemistry indicated acute reduced renal function which resolved with administration of IV fluids. Subsequently, he began to eat and drink small amounts and was discharged after a few days. However, a pattern of refusal to eat and drink developed, resulting in further six admissions to hospital with similar symptoms of dehydration and acute kidney injury over the following 6 months. During this time, his presentation deteriorated to the severity of behaviours described above. He also communicated via letters to his mother and typing on his iPad that he would like to die and return as a Labrador dog or as a superhero. On two occasions, during his admissions to the acute hospital, attempts were made to administer medication to him. However, on each occasion he refused and only one dose of fluoxetine was administered via his NG tube. He also refused administration of aripiprazole and no doses were given. Intramuscular administration of psychotropic medication did not seem proportional given the lack of evidence for medication being successful in PRS cases [4]. Given that he began to accept oral intake and made attempts to remove his NG tube, it was also not appropriate or practical to continue with administration of psychotropic medication via this route.

Our patient was born at full term by Caesarean section with no complications. The pregnancy was described as uneventful and there was no exposure to alcohol, tobacco, or illicit substances. He achieved his developmental milestones within a normal timeframe. No concerns were raised prior to starting nursery school (age 4 years). He was noted to be “different” to other children in his class and was diagnosed with ASD at the age of 6 years using Autism Diagnostic Observation Schedule (ADOS) and extensive interviews with his mother. A cognitive assessment, the Wechsler Intelligence Scale for Children (WISC-IV), was completed at age 6, which showed an IQ of 78, indicating a low normal intelligence (and therefore no intellectual disability).

In this case it is pertinent to consider the nature and extent of his preexisting ASD symptoms prior to his symptoms at presentation in 2016. In his records, he is described as struggling with understanding abstract questions, complex reasoning, and problem solving skills. He displayed formal speech at times and had difficulties with reciprocal conversation. He spoke quietly with deficits in prosody and struggled to contribute in class at school, but there was no history of muteness at the time of ASD diagnosis. He could misinterpret instructions and had a literal understanding of language. He showed avoidance of eye contact and limited facial expressions. He preferred to be alone, struggled with imaginative play, and did not initiate interaction with other children. He had specific interests in superheroes and dressing in these costumes. In younger childhood, he showed specific interest in vacuum cleaners and washing machines. He also was observed to make repetitive movements in the form of a “jerking” of his neck.

Since admission to the ward in July 2016, he made comparatively quick progress and was discharged into the community in November 2016. Initially he began to eat only a small amount of prepackaged food. He was reviewed by the dietician, who advised prescribing supplement drinks, which he initially accepted two of. The rationale of the admission to hospital was clearly explained to him and that, as long as he was not eating or drinking, he would need to be in hospital. We believe that this triggered his decision to begin to accept diet and fluids. He accepted a change in clothes and in his personal hygiene routine with the assistance of nursing staff. He made it clear to his mother (via written letters) that he wanted to be at home and that he believed that there was no need for him to be in hospital as he was accepting diet and fluids and had changed his clothes. He also attended his tribunal himself to appeal his detention under Section 2 of the Mental Health Act but did not communicate to the tribunal panel, verbally or otherwise.

He made steady progress with regard to dietary intake and gradually accepted full meals served on the ward. His engagement with nursing and allied therapy staff began to improve; he began to communicate with hand gestures (“thumbs up or down”) and use written notes. We therefore agreed to allow him to leave the ward to his family home at weekends. He also started to very quietly verbally communicate with his mother. He began to smile and laugh appropriately with staff and peers. However, he would hide his mouth with his fingers to avoid others seeing this. When asked if he would like to participate in activities, he declined, but when asked if he would, he engaged in a full timetable and appeared to enjoy some of the activities. We believe this is an indication of his preference to avoid activities and a symptom of his ASD. After a period of assessment, speech and language therapy (SALT) advised staff on the phrasing of questions to avoid confusion. Occupational therapy (OT) guided nursing staff, with whom he had established a better therapeutic relationship, with regard to reestablishing his daily skills including dressing and self-care.

He progressed to having day leave from the ward and started to attend a specialist school with support in the classroom to assist his communication and learning needs. Although he is not verbally communicating other than with his mother, we believe that his prognosis is good based on his current progress. We expect him to make a full recovery to his premorbid functioning with support in the community. It is difficult to comment upon what his ultimate level of communication will be like as his ASD is likely to have an impact upon this. However, given his young age, with specialist support for his ASD he has the potential to develop coping strategies. At discharge from hospital, he indicated no signs of distress with his level of communication and interaction (i.e., verbally with his mother). However, he will require further input from the community team to accurately assess his long term goals regarding verbal communication for the future.

## 3. Discussion

In the described case there are several interesting points to consider. Firstly, there is the issue of diagnosing Pervasive Refusal Syndrome in this clinical case. Due to the characteristics of his ASD, there were some coexisting features related to difficulties with verbal communication. Despite this, there were no periods of mutism prior to this presentation. As discussed, there can be features of food refusal or selectivity, refusal of engagement in personal hygiene, and mutism or lack of communication with others in ASD. However, at what point would this be considered to be best explained by a diagnosis of ASD, and when would a diagnosis of Pervasive Refusal Syndrome be more likely? We believe that a diagnosis of Pervasive Refusal Syndrome better describes the symptoms than ASD alone when the following present together: a significant change in functioning, significant level of severity (e.g., seven admissions to hospital, for NG tube feeding, not taking off coat, and total mutism), and an overt trigger that is perceived as stressful. Nonetheless, there are clearly potential difficulties in making a distinction.

Another consideration is that of a differential diagnosis of catatonia in this case. There have been discussions in the literature regarding the validity of a diagnosis of PRS and whether this may be a manifestation of catatonia [14, 15]. However, whilst there was clear evidence of mutism and some evidence of agitation and negativism, these did not meet the DSM-5 diagnostic criteria for catatonia [16]. He demonstrated a level of physical agitation towards his stepfather and siblings yet not towards his mother or members of staff, indicating a variance of this behaviour and clear response to external stimuli. His opposition was representative of an angry response to certain demands made by others. There was variation in the level of negativism, according to who had made the demand and its nature, demonstrating that this was in response to external stimuli. There was also no evidence of movement disorders, stupor, or echolalia.

This leads to the next consideration. Given the overlap of domains of presenting behaviour in ASD and PRS as discussed, it is important to consider if there could be an association or interaction between the two conditions. Is it possible that a diagnosis of ASD could predispose to a presentation of behaviours that would be collectively diagnosed as PRS? A trigger or event perceived as traumatic in children with symptoms or traits of ASD may be more likely to lead to decline in functions in various domains. The perception of the incident may also be more likely to be interpreted as traumatic in this population. The current knowledge of the demographics of PRS indicates that the presentation is more common in girls than boys. It has been established, however, that ASD is underdiagnosed in girls [17]. It could be possible that prior to the presentation of PRS in these girls they have functioned well and ASD traits have been less obvious, hence undiagnosed.

The other question that this case raises is what impact a comorbid presentation of ASD has on the progress and prognosis of PRS. Our case showed significant progress and was discharged from our ward after 4 months, which is significantly quicker than the mean of over 12 months (from previous literature). It is possible that the ASD symptoms such as literal thinking and concrete processing have actually aided in speeding up the recovery process. The clinical team working with our case quickly found that he responded very well to rules, boundaries, and clear consequences of behaviour. It should also be noted that the clinical team working with him were skilled in a Positive Behavioural Support (PBS) approach. We therefore gathered a significant amount of high quality data about his behaviours, which guided the therapeutic approach. He began accepting diet and fluids on the ward and returned from leave, as it was made clear that he would have to stay in hospital and be brought back by police should he attempt to abscond. The interplay between ASD and PRS remains unknown at present, but we feel that using this style of behavioural and consequences approach with children with PRS and ASD could prevent further admissions and improve the prognosis and recovery of PRS.

## Data Availability

It is not applicable as the article describes a clinical case. The information discussed in the background and any statements made are all individually referenced; hence the data surrounding any points discussed can be accessed directly through these articles.
